# The Army Examining Board

**Published:** 1901-04

**Authors:** 


### THE ARMY EXAMINING BOARD.

THIS board has organized at Washington with Dr. John S. Marshall as president, and upon another page will be found an announcement which came too late for the March number, but will serve a purpose by giving the dental profession an idea of the methods proposed to be adopted by the board in the examination of applicants for positions. While these examinations are, at this writing, March 8, fully under way, it is presumed that many who would gladly accept the opportunity have not been made aware of the fact. If any public notice has been made by said board of the proposed examinations, it has escaped the observation of the writer. The twenty-seven men to be appointed will have a serious task before them, for to these belongs the duty of proving to the critical military authorities that all that has been said of the value of dentistry to the army is absolutely true. The general dental profession will also watch their work critically, and if they fail to measure up to a high standard, it will mean a serious disappointment; and not only that, but it will lower the entire dental profession in the estimation of those whose good opinion is most to be valued.

The men in dentistry have never asked that Congress should pass this bill from selfish motives. It was affirmed, and was well supported by evidence, that the army was suffering from lack of just the aid that well-trained dentists could give, and it was for the benefit of the men that the bill was finally adopted and received the signature of the President.

In the March number a brief allusion was made to the selection of the men to compose the examining board. These three appointments leave but twenty-seven men to be selected to serve as contract dental surgeons. It is supposed the army will be recruited up to the maximum number,—that is, one hundred thousand. This will give a clientele of four thousand men to each dental surgeon. This of itself is sure to result in failure. No man living can perform dental work properly for that number. If he were to work eight hours a day, Sundays included, he would have waited on something over four thousand in a year and a half; that is, operating only once for a single individual.

The bill, as passed, is absurd, but it was something to get one through, and it is possible to have it amended in the future, when an effort should be made to secure two dental surgeons to a regiment; or, if that be not possible, at least one for a thousand men.

The bill in its present shape, and the appointments made under it, have been a serious disappointment to those who have labored for years to accomplish its passage. The great desire of the best class of dentists was that the highest type of men should receive the initial appointments. It was further hoped that politics would be eliminated, at least for the time being.

The effort was made throughout the United States to present only the best representative men that could be found willing to accept the chief positions. It was naturally feared that it would be difficult to find that character of men willing to serve on the meagre salary provided in the bill. It was well understood that whoever did elect to represent the dental profession in the army, it would be at great pecuniary sacrifice as well as comfort. It was, therefore, something of a surprise that a number of men known to be well qualified offered their services, and the dentists of the country were not slow to appreciate the fact that this sacrifice was worthy of the men, and, recognizing this, they received cordial endorsement from all sections. It was supposed that the heads of the government appreciated the full measure of responsibility devolving on their selections, and in making these appointments only the highest good of the army would be taken into consideration.

The blow came when it was officially announced that three had been appointed. One man of the three required no endorsement. He was known everywhere as fully equal to all demands; some few knew another by name, but the other was by the writer entirely unknown. This latter appointment cannot be regarded complacently, as it is felt to be entirely at variance with the wishes of the entire dental fraternity that only men well known should fill these important positions.

The dental profession expected, nay, more, practically demanded, that Dr. Williams Donnally, of Washington, should be a member of this board. Throughout the long and vexatious period in which the several bills presented to Congress were from time to time held up he was ever the earnest and faithful worker, spending liberally of his own means and time that the dental profession might not be disappointed at last. He knew that the National Dental Association would hold him largely responsible for the success or failure of this measure. He met the responsibility with a courage and persistency that have throughout this trying period claimed the admiration of the writer. It was well known that this work of Dr. Donnally was truly unselfish. He had an ambition, an honorable one, to help inaugurate this movement and place it upon a permanent basis, and the dental profession felt that this desire was worthy of the man, and did not for a moment anticipate that the appointing power would select any one who failed to represent the dentistry of the United States.

The reason said to have been given for thus casting Dr. Donnally outside the pale of appointments may have force with the party originating it, but to the writer it seems unworthy an officer of the government. The opinion was, simply, "That no one who had been influential in securing legislation should personally profit by the legislation when enacted."

This, of course, made Dr. Donnally's appointment an impossibility. There was no objection, apparently, to men who had worked for *themselves* and had labored hard to secure all the political influence possible, both at Washington and elsewhere.

If men could be selected to fill these and other important positions because of their marked ability, a course pursued by all well-regulated corporations, there would be a very different story told throughout this "boss"-ridden country.

The writer is well aware that Dr. Donnally would be the very last man to desire his personal feelings ventilated upon the pages of a public journal, but it is felt he must add this sacrifice to those he has previously made for his profession, in order that the dentists of the country may understand the situation. It is the most lamentable ending of an heroic struggle that dentistry has known in many years.

This profession owes much to another man, not within its borders, Surgeon-General George M. Sternberg, who has from the first fully appreciated the importance of having the right man in the right place; and while his powerful influence has been exerted on behalf of the bill, and without it it probably could not have been passed, he necessarily had no prominent part in the appointments. His name is, therefore, merely mentioned to voice the grateful feeling the writer is well aware exists.

The thanks of the dental profession should be gratefully tendered to Senator Edward W. Pettus, of Alabama, the father of the bill in the Senate, and to Hon. P. J. Otey for his great work in the House. This labor was given unselfishly, and the National Dental Association should, at its next meeting in August, remember these facts and act accordingly.

It is a great satisfaction to feel that the presidency of the board of examiners is centred in Dr. Marshall, of Chicago. There is a confidence felt in his ability that will go far to minimize the disappointment alluded to, but it cannot dissipate the unpleasant feeling existing.

In the opinion of the writer a concerted action should be had during the sessions of the National Dental Association, if not before, to protest against any appointments upon mere political recommendation. These should follow upon examination made by a temporary commission. The government could well afford to set an example in this respect to the several States, where political influence is fast destroying all respect for State boards. Further than this, it does seem that an immediate protest should go to the appointing power, and, if necessary, to the President, asking that all future selections be made upon the endorsement of the national organization.

It is presumed it is too late to attempt anything on behalf of the one man the dentists of the country desired on that board, but something of value may be done in this direction in the near future if proper efforts be made.

The responsibility of failure of this effort to establish dental operations in the army should be placed where it properly belongs, —upon the shoulders of Congress. This body has made its success almost an impossibility. It is probable that this result was not intended, for the average layman has but a limited conception of the value of dental services, and especially so where the men in the army are concerned. All that is usually expected of dentists in such positions is to extract teeth and "stop toothache." The pathological conditions that so militate against efficient service are rarely, if ever, considered in connection with dental work.

There is no desire to be a prophet of ill-omen in this connection, but it does seem to the writer that the non-success of this experiment is fully assured under present arrangements. The men selected, however able or earnest, cannot fulfil the duties required of them. When this is demonstrated and a demand is made in Congress for the repeal of the act creating contract dental surgeons, let the blame for the failure rest on Congress, and not upon dentistry or upon the men who so faithfully worked until the act creating dental surgeons in the army was finally passed.

